# Frequency and Clinical Correlates of Sarcopenia in Patients With Metabolic Dysfunction-Associated Steatotic Liver Disease

**DOI:** 10.7759/cureus.116554

**Published:** 2026-09-19

**Authors:** Tahseer Khan, Syeda Batool Tirmizi, Reena Kumari, Omar Naeem, Abida Bukhari, Ayesha Ali Qureshi, Usman Khan, Muhammad Irfan Jamil

**Affiliations:** 1 Internal Medicine, Dow University Hospital, Karachi, PAK; 2 Internal Medicine, Liaquat University of Medical and Health Sciences, Jamshoro, PAK; 3 General Physician, Sulaiman Medical Center, Al Ain, ARE; 4 Internal Medicine, King Edward Medical University, Lahore, PAK; 5 Infectious Disease, University Hospital Waterford, Waterford, IRL; 6 Internal Medicine, Lahore Medical and Dental College, Lahore, PAK; 7 General Physician, Nishtar Medical College and Hospital, Multan, PAK; 8 General Physician, Lancashire Teaching Hospitals, Preston, GBR; 9 Integrative Medicine, Shaikh Khalifa Bin Zayed Al Nahyan Medical College, Lahore, PAK; 10 Nephrology, Lahore General Hospital, Lahore, PAK; 11 Gastroenterology, King Edward Medical University, Lahore, PAK

**Keywords:** insulin resistance, liver fibrosis, metabolic dysfunction-associated steatotic liver disease, sarcopenia, transient elastography

## Abstract

Introduction

Sarcopenia may occur in people with metabolic dysfunction-associated steatotic liver disease; however, the condition's frequency and correlation with liver fibrosis vary by group. Patients with reduced muscle mass and strength may also have a higher metabolic burden and more severe liver involvement. Because these relationships remain inconsistent across available studies, this study aimed to estimate the prevalence of sarcopenia in patients with metabolic dysfunction-associated steatotic liver disease (MASLD) and examine its association with clinical findings, metabolic profile, body composition, and liver-related features.

Methods

A total of 276 people suffering from MASLD were recruited consecutively from a tertiary care hospital for this cross-sectional study. Sarcopenia was diagnosed when low appendicular skeletal muscle mass was present together with either low handgrip strength or reduced physical performance. Severe sarcopenia was defined by the presence of low muscle mass, low muscle strength, and reduced physical performance. Liver steatosis was assessed using the Control Attenuation Parameter (CAP), and liver stiffness measurement (LSM) was utilized to evaluate fibrosis. The differences between patients with and without sarcopenia were assessed. A binary logistic regression was employed, taking into account relevant clinical parameters, to ascertain if sarcopenia and fibrosis were significantly correlated.

Results

Among 276 patients, the prevalence of sarcopenia was 23.2% (64/276; 95% confidence interval (CI), 18.6-28.5%). Sarcopenia patients were older (54.9 ± 10.6 vs. 48.1 ± 11.1 years; p < 0.001), more likely to have diabetes mellitus (56.2% vs. 32.5%; p < 0.001) and hypertension (57.8% vs. 35.8%; p = 0.002), and less likely to be physically active (21.9% vs. 53.3%; p < 0.001). Sarcopenia patients exhibited greater median LSM (8.4 (6.0-10.6) vs. 6.6 (5.3-8.1) kPa; p < 0.001) and CAP (311.7 ± 28.7 vs. 297.8 ± 34.1 dB/m; p = 0.001). Significant fibrosis was present in 51.6% of patients with sarcopenia compared to 26.4% of patients without sarcopenia (p < 0.001). After adjustment for age, sex, body mass index, and diabetes, sarcopenia remained statistically associated with significant fibrosis (adjusted odds ratio (OR) 2.03, 95% CI 1.10-3.77; p = 0.024).

Conclusion

Sarcopenia remained statistically associated with significant fibrosis after adjustment for relevant clinical factors; however, further prospective studies are needed to determine whether assessment of muscle status has clinical utility in patients with MASLD.

## Introduction

Metabolic dysfunction-associated steatotic liver disease (MASLD), which is identified when hepatic steatosis and at least one cardiometabolic risk factor are present, has taken the place of non-alcoholic fatty liver disease [1]. MASLD encompasses a spectrum ranging from hepatic steatosis to metabolic dysfunction-associated steatohepatitis (MASH) and may be accompanied by varying degrees of liver fibrosis, including cirrhosis [2]. This condition is typically seen in obese people or those with type 2 diabetes, hypertension, and dyslipidemia, demonstrating its close relation to metabolic health, rather than the liver only [3]. Recognizing factors associated with a higher disease burden may help identify patients who need more careful clinical assessment.

Skeletal muscle plays an essential function in the use of glucose, as well as the sensitivity to insulin and balance of energy. Loss of muscle mass and strength may occur alongside insulin resistance, excess adiposity, and chronic inflammation, which are also common in MASLD [4]. Sarcopenia includes reduced muscle quantity or quality with loss of muscle strength and, in more severe cases, reduced physical performance. A recent pooled analysis reported sarcopenia in 23.5% of patients with MASLD and found an association with poorer clinical outcomes, although prevalence varied widely because different populations and methods were used to assess muscle status [5].

Recent original studies support an association between sarcopenia and more severe liver disease, but findings are not uniform. Significant fibrosis was seen in 18.3% of patients with both nonalcoholic fatty liver disease (NAFLD) and sarcopenia in a US study of 2,422 people, compared to 9.9% of patients with NAFLD without sarcopenia [6]. Long-term population data also showed higher all-cause mortality and liver fibrosis when metabolic fatty liver disease and sarcopenia occurred together [7]. In contrast, a biopsy-based study found that myosteatosis, rather than sarcopenia, was related to early steatohepatitis and fibrosis progression [8]. More recent data from 821 patients from the United States and 3,405 patients from China with MASLD again showed greater fibrosis risk among patients with sarcopenia [9].

These differences show that the frequency and clinical importance of sarcopenia in MASLD may depend on age, metabolic status, body composition, and the method used to define muscle loss. Sarcopenia in patients with MASLD may be associated with a greater metabolic and liver-related burden beyond that reflected by body weight alone. This study aimed to evaluate the degree of sarcopenia in people with MASLD and to determine how it connected to their liver-related, metabolic, and clinical body composition traits.

## Materials and methods

Between July 2025 and June 2026, this cross-sectional study was carried out in the gastroenterology department of Mayo Hospital in Lahore, Pakistan. The Institutional Review Board, King Edward Medical University/Mayo Hospital, Lahore, gave its approval (Approval No.: 145/RC/KEMU) before the first patient was admitted. Written agreement was obtained before any study-related examination, and patients who met the study's conditions were included in the order in which they were presented. MASLD was defined by the presence of hepatic steatosis together with at least one cardiometabolic risk factor. These included excess adiposity, impaired glucose regulation, high blood pressure, elevated triglyceride levels, or low high-density lipoprotein cholesterol, according to the multisociety definition of MASLD [1,2]. Hepatic fat was assessed using vibration-controlled transient elastography. A controlled attenuation parameter (CAP) value of ≥248 dB/m was used to define hepatic steatosis. This cutoff was selected on the basis of the individual-patient-data meta-analysis by Karlas et al., which identified 248 dB/m as the optimal threshold for detecting ≥S1 hepatic steatosis [10]. Patients were ruled out if a different reason for persistent liver disease was evident, including viral hepatitis, autoimmune or chronic cholestatic liver disease, Wilson disease, hemochromatosis, or alcohol consumption over the limit allowed with MASLD. Patients with decompensated cirrhosis, defined by current or previous ascites, variceal bleeding, hepatic encephalopathy, or jaundice [11]; advanced chronic kidney disease, defined as an estimated glomerular filtration rate <30 mL/min/1.73 m² [12]; severe heart failure, defined as New York Heart Association functional class III or IV [13]; active malignancy; chronic inflammatory disease; neuromuscular illness; or another condition expected to cause major loss of skeletal muscle were also not eligible. The same applied to patients who had undergone major surgery, remained immobile for a prolonged period, developed a serious acute illness, or had been admitted to hospital during the previous three months. Pregnancy, limb amputation, clinically evident ascites or marked peripheral edema on physical examination, and the presence of an implanted electronic device that could interfere with bioelectrical impedance measurement were additional reasons for exclusion. Patients with ascites or marked peripheral edema were excluded before bioelectrical impedance analysis because fluid retention could affect the estimation of body composition and muscle mass.

Using an estimated prevalence of sarcopenia/low skeletal muscle mass of 23.4% among patients with fatty liver disease, the sample size was calculated for estimation of a single population proportion [14]. At a 95% confidence level and 5% absolute precision, the calculated sample size was 275.4 and was rounded to 276 patients. This calculation addressed the prevalence objective of the study. The association between sarcopenia and significant fibrosis was subsequently assessed using logistic regression. In the final sample, significant fibrosis was present in 89 patients, and the adjusted model included five parameters (sarcopenia, age, sex, BMI, and diabetes mellitus), corresponding to approximately 17.8 fibrosis events per model parameter, providing an adequate number of outcome events for the planned multivariable analysis:

\( n = \frac{Z^2 p(1-p)}{d^2} = \frac{(1.96)^2(0.234)(0.766)}{(0.05)^2} = 275.4 \)

where n represents the minimum required sample size; Z is the standard normal deviate corresponding to a 95% confidence level (1.96); p is the anticipated prevalence of sarcopenia (23.4%, or 0.234); and d is the desired absolute precision (5%, or 0.05). The calculated minimum sample size was 275.4, which was rounded up to 276 patients.

A structured proforma was used to collect the data. Age and sex, height, weight, body mass index (BMI), BMI category, waist circumference, central obesity, length of MASLD, systolic and diastolic blood pressure, diabetes, dyslipidemia, hypertension, metabolic syndrome, smoking, and physical activity were among the demographic and clinical factors. BMI was calculated as weight in kilograms divided by height in metres squared (kg/m²) and was classified according to World Health Organization criteria as normal (18.5-24.9 kg/m²), overweight (25.0-29.9 kg/m²), and obesity (≥30.0 kg/m²). Metabolic syndrome was defined by the presence of at least three of the following: waist circumference ≥90 cm in men or ≥80 cm in women, triglycerides ≥150 mg/dL, HDL-C <40 mg/dL in men or <50 mg/dL in women, blood pressure ≥130/85 mmHg or use of antihypertensive medication, and fasting glucose ≥100 mg/dL or previously diagnosed diabetes mellitus. Fasting glucose, hemoglobin A1c (HbA1c), fasting insulin, total cholesterol and triglycerides, HDL cholesterol (HDL-C) and low-density lipoprotein cholesterol (LDL-C), aspartate aminotransferase (AST), alanine aminotransferase (ALT), gamma-glutamyl transferase (GGT), alkaline phosphatase (ALP), bilirubin, platelet count, albumin-to-creatinine ratio, high-sensitivity CRP (hs-CRP), and ferritin were all included in the laboratory assessment. The homeostatic model assessment of insulin resistance (HOMA-IR) was calculated as fasting glucose (mg/dL) × fasting insulin (µIU/mL)/405. The proportion of body fat and visceral fat areas were included in the body composition assessment. Appendicular muscle mass (ASM) and the appendicular skeletal muscle index (ASMI) were computed by dividing ASM by the squared height.

The Asian Working Group for Sarcopenia 2019 framework was used to evaluate sarcopenia [15]. The InBody 770 Body Composition Analyzer (InBody Co., Ltd., Seoul, Republic of Korea) was used to calculate appendicular muscle mass by bioelectrical impedance analysis. The Jamar Plus+ digital hand dynamometer (Jamar, Performance Health, USA) was used to measure handgrip strength. Physical performance was evaluated using a five-time chair stand test and usual gait speed. Low muscle mass was defined as an appendicular skeletal muscle index of <7.0 kg/m² for men and <5.7 kg/m² for women. Low muscle strength was defined as handgrip strength <28 kg for men or <18 kg for women. Reduced physical performance was defined as a usual gait speed <1.0 m/s or a five-time chair stand time ≥12 seconds [15]. Possible sarcopenia was defined as low muscle strength and/or reduced physical performance without meeting the criteria for sarcopenia. Sarcopenia was defined as low muscle mass together with either low muscle strength or reduced physical performance, while severe sarcopenia required low muscle mass, low muscle strength, and reduced physical performance [15]. SARC-F was used as a screening tool, and a score ≥4 was considered a positive screening result [16]. Steatosis of the liver and liver stiffness were measured by transient elastography using the controlled attenuation parameter and liver stiffness measurement (LSM), respectively. An LSM value ≥8.0 kPa was used to define LSM-based significant fibrosis [6]. For this study, the term “significant fibrosis” refers to this noninvasive LSM-based definition. The same measurement approach and diagnostic thresholds were used for all participants.

IBM SPSS Statistics for Windows, version 26 (IBM Corp., Armonk, N.Y., USA) was used to analyze the data. The Shapiro-Wilk test was used to evaluate the skewness of continuous variables. The mean ± standard deviation was used to represent non-skewed data, while the median with interquartile range was used to portray skewed data. Depending on the data distribution, either the independent-samples t-test or the Mann-Whitney U test was used to test for continuous variables. Fisher's exact test or Pearson's chi-square were used to compare categorical variables, which were displayed as frequencies and percentages. Factors associated with significant fibrosis were examined using univariable binary logistic regression, with LSM-based significant fibrosis (LSM ≥8.0 kPa) as the outcome variable and sarcopenia as the main exposure variable. The multivariable model included age, sex, BMI, and diabetes mellitus. The 95% confidence intervals for the crude and adjusted odds ratios were computed and presented. In every two-sided statistical test, P < 0.05 was considered statistically significant.

## Results

The final study comprised 276 patients with steatotic liver disease linked to metabolic dysfunction. The prevalence of sarcopenia was 23.2% (95% confidence interval 18.6-28.5%) in 64 patients, while it was absent in 212 patients. The study group as a whole had an average age of 49.7 ± 11.3 years, with 163 participants (59.1%) being male. Patients with sarcopenia had a higher mean body mass index (29.8 ± 3.6 vs. 28.1 ± 3.4 kg/m²; p < 0.001) and were older (54.9 ± 10.6 vs. 48.1 ± 11.1 years; p < 0.001). Among the patients, 56.2% with sarcopenia and 32.5% without sarcopenia had diabetes mellitus (p < 0.001), while 57.8% and 35.8% of patients, respectively, had hypertension (p = 0.002). The sarcopenia group reported less frequent physical activity (21.9% vs. 53.3%; p < 0.001). Sex, waist circumference, central obesity, length of MASLD, systolic blood pressure, dyslipidemia, metabolic syndrome, and current smoking did not show any significant group differences (Table 1).

**Table 1:** Baseline demographic and clinical characteristics stratified by sarcopenia status (n = 276). Values are presented as mean ± standard deviation (SD), median (interquartile range (IQR)), and number (percentage). Welch's independent-samples t-test was used for approximately normally distributed continuous variables, the Mann-Whitney U test for non-normally distributed continuous variables, and the Pearson chi-square test for categorical variables. The test statistic and degrees of freedom (df), where applicable, are reported in separate columns. Cramer's V is reported with Pearson chi-square tests as the effect-size measure. BMI: body mass index; MASLD: metabolic dysfunction-associated steatotic liver disease.

Variable	Overall (n = 276)	Sarcopenia group (n = 64)	No sarcopenia group (n = 212)	Test statistic	df	p-value
Age (years), mean ± SD	49.7 ± 11.3	54.9 ± 10.6	48.1 ± 11.1	t=4.45	108.5	<0.001
Sex, n (%)				χ²=1.94 V=0.08	1	0.164
Male	163 (59.1)	33 (51.6)	130 (61.3)			
Female	113 (40.9)	31 (48.4)	82 (38.7)			
Body mass index (kg/m²), mean ± SD	28.5 ± 3.5	29.8 ± 3.6	28.1 ± 3.4	t=3.44	100.1	<0.001
BMI category, n (%)				χ²=10.01 V=0.19	2	0.007
Normal	40 (14.5)	2 (3.1)	38 (17.9)			
Overweight	147 (53.3)	35 (54.7)	112 (52.8)			
Obesity	89 (32.2)	27 (42.2)	62 (29.2)			
Waist circumference (cm), mean ± SD	94.0 ± 8.0	95.3 ± 9.2	93.6 ± 7.5	t=1.38	89.9	0.171
Central obesity, n (%)	236 (85.5)	57 (89.1)	179 (84.4)	χ²=0.85 V=0.06	1	0.357
MASLD duration (years), median [IQR]	3.8 [2.1-6.0]	4.3 [2.2-6.7]	3.7 [2.1-5.9]	U=7374.5	—	0.292
Systolic blood pressure (mmHg), mean ± SD	128.3 ± 13.8	129.9 ± 14.5	127.8 ± 13.6	t=1.02	99.2	0.312
Diastolic blood pressure (mmHg), mean ± SD	80.1 ± 8.8	82.7 ± 8.8	79.3 ± 8.7	t=2.67	103.2	0.009
Diabetes mellitus, n (%)	105 (38.0)	36 (56.2)	69 (32.5)	χ²=11.72 V=0.21	1	<0.001
Hypertension, n (%)	113 (40.9)	37 (57.8)	76 (35.8)	χ²=9.81 V=0.19	1	0.002
Dyslipidemia, n (%)	144 (52.2)	37 (57.8)	107 (50.5)	χ²=1.06 V=0.06	1	0.303
Metabolic syndrome, n (%)	201 (72.8)	50 (78.1)	151 (71.2)	χ²=1.18 V=0.07	1	0.277
Current smoking, n (%)	69 (25.0)	15 (23.4)	54 (25.5)	χ²=0.11 V=0.02	1	0.742
Physically active, n (%)	127 (46.0)	14 (21.9)	113 (53.3)	χ²=19.55 V=0.27	1	<0.001

According to the sarcopenia-stage classification, 186 patients (67.4%) had no sarcopenia, 26 (9.4%) had possible sarcopenia, 46 (16.7%) had sarcopenia, and 18 (6.5%) had severe sarcopenia. Patients classified as having sarcopenia had significantly lower appendicular skeletal muscle mass (ASMI, handgrip strength, calf circumference, and gait speed, together with a longer chair-stand time and higher SARC-F score. A SARC-F score ≥4 was present in 41/64 (64.1%) patients with sarcopenia compared with 5/212 (2.4%) patients without sarcopenia (p < 0.001). Patients with sarcopenia also showed a less favorable metabolic profile. Median HbA1c was higher in the sarcopenia group (6.5% (IQR 5.7-7.2) vs. 5.8% (5.3-6.5); p < 0.001), together with higher fasting insulin (21.9 (16.3-32.5) vs. 17.9 (11.9-25.1) µIU/mL; p = 0.003) and HOMA-IR (6.71 (3.97-10.64) vs. 4.91 (2.86-7.24); p < 0.001). Triglyceride levels were also higher (181.0 (142.0-235.2) vs. 157.0 (122.5-196.0) mg/dL; p = 0.004). Median ALT was higher in patients with sarcopenia (50.0 vs. 43.0 U/L; p = 0.031), while platelet count was lower (227.5 ± 47.3 vs. 244.1 ± 48.2 ×10⁹/L; p = 0.016) and eGFR was lower (98.1 ± 11.5 vs. 102.3 ± 11.9 mL/min/1.73 m²; p = 0.012). Median hs-CRP was significantly higher among patients with sarcopenia (3.54 (2.41-5.04) vs. 2.34 (1.65-3.54) mg/L; p < 0.001). Other lipid and liver biochemical parameters were not significantly different between groups (Table 2).

**Table 2:** Laboratory and metabolic parameters of the study population, stratified by sarcopenia status (n = 276). Values are presented as mean ± SD or median (IQR), according to distribution. P-values were derived using the independent-samples t-test or Mann-Whitney U test, as appropriate. HbA1c: glycated hemoglobin; HOMA-IR: homeostatic model assessment of insulin resistance; HDL-C: high-density lipoprotein cholesterol; LDL-C: low-density lipoprotein cholesterol; AST: aspartate aminotransferase; ALT: alanine aminotransferase; GGT: gamma-glutamyl transferase; ALP: alkaline phosphatase; eGFR: estimated glomerular filtration rate; hs-CRP: high-sensitivity C-reactive protein.

Variable	Overall (n = 276)	Sarcopenia group (n = 64)	No sarcopenia group (n = 212)	Test statistic	df	p-value
Fasting glucose (mg/dL), median (IQR)	111.0 (93.0-131.2)	117.0 (96.0-136.2)	109.5 (92.8-129.0)	U=7823.5	—	0.063
HbA1c (%), median (IQR)	5.9 (5.4-6.7)	6.5 (5.7-7.2)	5.8 (5.3-6.5)	U=8979.5	—	<0.001
Fasting insulin (µIU/mL), median (IQR)	18.9 (12.6-26.4)	21.9 (16.3-32.5)	17.9 (11.9-25.1)	U=8465.5	—	0.003
HOMA-IR, median (IQR)	5.32 (3.09-7.80)	6.71 (3.97-10.64)	4.91 (2.86-7.24)	U=8651.0	—	<0.001
Total cholesterol (mg/dL), mean ± SD	204.2 ± 31.4	207.0 ± 32.2	203.3 ± 31.2	t=0.81	101.5	0.419
Triglycerides (mg/dL), median (IQR)	162.5 (127.0-203.2)	181.0 (142.0-235.2)	157.0 (122.5-196.0)	U=8374.5	—	0.004
HDL-C (mg/dL), mean ± SD	45.0 ± 9.5	44.0 ± 10.5	45.4 ± 9.2	t=-0.91	93.8	0.366
LDL-C (mg/dL), mean ± SD	124.5 ± 27.1	124.1 ± 28.5	124.6 ± 26.8	t=-0.12	99.0	0.904
AST (U/L), median (IQR)	34.0 (27.0-43.2)	36.5 (30.0-46.2)	34.0 (27.0-42.0)	U=7622.0	—	0.134
ALT (U/L), median (IQR)	45.0 (35.0-57.0)	50.0 (36.2-63.2)	43.0 (35.0-56.0)	U=7992.5	—	0.031
GGT (U/L), median (IQR)	45.5 (32.8-64.2)	46.0 (35.8-70.5)	45.5 (32.0-64.0)	U=7020.0	—	0.674
ALP (U/L), mean ± SD	89.9 ± 21.0	94.2 ± 21.7	88.6 ± 20.7	t=1.83	100.0	0.071
Total bilirubin (mg/dL), median (IQR)	0.71 (0.59-0.82)	0.70 (0.58-0.83)	0.71 (0.60-0.82)	U=6682.0	—	0.856
Albumin (g/dL), mean ± SD	4.18 ± 0.27	4.16 ± 0.28	4.18 ± 0.27	t=-0.56	99.6	0.579
Platelet count (×10⁹/L), mean ± SD	240.3 ± 48.4	227.5 ± 47.3	244.1 ± 48.2	t=-2.45	105.6	0.016
Serum creatinine (mg/dL), mean ± SD	0.86 ± 0.17	0.84 ± 0.16	0.87 ± 0.17	t=-1.36	107.7	0.175
eGFR (mL/min/1.73 m²), mean ± SD	101.3 ± 11.9	98.1 ± 11.5	102.3 ± 11.9	t=-2.55	107.4	0.012
hs-CRP (mg/L), median (IQR)	2.52 (1.77-3.95)	3.54 (2.41-5.04)	2.34 (1.65-3.54)	U=9237.5	—	<0.001
Ferritin (ng/mL), median (IQR)	152.5 (104.8-238.8)	156.5 (124.8-276.2)	150.5 (104.0-230.0)	U=7553.5	—	0.169

According to the sarcopenia-stage classification, 186 patients (67.4%) had no sarcopenia, 26 (9.4%) had possible sarcopenia, 46 (16.7%) had sarcopenia, and 18 (6.5%) had severe sarcopenia. Patients classified as having sarcopenia had significantly lower appendicular skeletal muscle mass (ASMI, handgrip strength, calf circumference, and gait speed, together with a longer chair-stand time and higher SARC-F score. A SARC-F score ≥4 was present in 41/64 (64.1%) patients with sarcopenia compared with 5/212 (2.4%) patients without sarcopenia (p < 0.001). The hepatic profile also differed between groups. The mean controlled attenuation parameter was higher in patients with sarcopenia (311.7 ± 28.7 vs. 297.8 ± 34.1 dB/m; p = 0.001), while median liver stiffness was 8.4 (6.0-10.6) kPa compared with 6.6 (5.3-8.1) kPa among patients without sarcopenia (p < 0.001). Significant fibrosis was present in 33/64 (51.6%) patients with sarcopenia compared with 56/212 (26.4%) patients without sarcopenia (p < 0.001) (Table 3).

**Table 3:** Body composition, sarcopenia-related measures, and liver-related parameters according to sarcopenia status (n = 276). Values are presented as mean ± SD, median (IQR), or number (percentage). P-values were derived using the independent-samples t-test, Mann-Whitney U test, or Pearson chi-square test, as appropriate. ASMI: appendicular skeletal muscle index; SARC-F: Strength, Assistance with walking, Rise from a chair, Climb stairs, and Falls; CAP: controlled attenuation parameter.

Variable	Overall (n = 276)	Sarcopenia group (n = 64)	No sarcopenia group (n = 212)	Test statistic	df	p-value
Body fat (%), mean ± SD	31.1 ± 7.2	34.4 ± 6.9	30.0 ± 7.0	t=4.44	105.1	<0.001
Visceral fat area (cm²), mean ± SD	119.2 ± 30.9	129.5 ± 35.1	116.1 ± 28.9	t=2.78	90.3	0.007
Appendicular skeletal muscle mass (kg), mean ± SD	20.2 ± 4.5	16.5 ± 3.6	21.3 ± 4.2	t=-9.11	120.5	<0.001
Appendicular skeletal muscle index (kg/m²), mean ± SD	7.25 ± 1.31	5.95 ± 0.75	7.64 ± 1.18	t=-13.60	164.0	<0.001
Handgrip strength (kg), mean ± SD	28.1 ± 7.7	20.9 ± 5.3	30.3 ± 6.9	t=-11.64	134.9	<0.001
Calf circumference (cm), mean ± SD	35.2 ± 1.9	34.2 ± 2.1	35.4 ± 1.8	t=-4.25	93.9	<0.001
Gait speed (m/s), mean ± SD	1.15 ± 0.11	1.07 ± 0.10	1.18 ± 0.10	t=-7.58	103.4	<0.001
Five-time chair stand (seconds), mean ± SD	9.4 ± 1.5	10.1 ± 1.5	9.2 ± 1.5	t=4.09	104.4	<0.001
SARC-F score, median (IQR)	2.0 (1.0-3.0)	4.0 (3.0-5.0)	1.0 (0.0-2.0)	U=12842.5	—	<0.001
SARC-F ≥4, n (%)	46 (16.7)	41 (64.1)	5 (2.4)	χ²=134.76 V=0.70	1	<0.001
Controlled attenuation parameter (dB/m), mean ± SD	301.0 ± 33.4	311.7 ± 28.7	297.8 ± 34.1	t=3.26	121.6	0.001
Liver stiffness measurement (kPa), median (IQR)	6.9 (5.4-8.6)	8.4 (6.0-10.6)	6.6 (5.3-8.1)	U=8871.5	—	<0.001
Significant fibrosis, n (%)	89 (32.2)	33 (51.6)	56 (26.4)	χ²=14.23 V=0.23	1	<0.001

In the univariable analysis, patients with sarcopenia had nearly threefold higher odds of LSM-defined significant fibrosis than those without sarcopenia (OR 2.97, 95% CI 1.66-5.28; p < 0.001). Significant fibrosis was also associated with diabetes mellitus, older age, higher body mass index, and physical inactivity in univariable analysis. The multivariable model included sarcopenia as the main exposure together with age, sex, body mass index, and diabetes mellitus. After adjustment, sarcopenia remained statistically associated with LSM-defined significant fibrosis (adjusted OR 2.03, 95% CI 1.10-3.77; p = 0.024). Each 10-year increase in age was associated with higher odds of LSM-defined significant fibrosis (adjusted OR 1.37, 95% CI 1.05-1.78; p = 0.020). The association with body mass index was attenuated and was not statistically significant after adjustment (adjusted OR 1.51 per 5 kg/m²; 95% CI 1.00-2.29; p = 0.053). Neither diabetes mellitus nor sex was statistically significant in the adjusted model. Overall, the model test was significant (likelihood-ratio χ²(5) = 28.76; p < 0.001). The Nagelkerke R² was 0.138, the area under the receiver operating characteristic curve was 0.687, and the Hosmer-Lemeshow test was non-significant (p = 0.344) (Table 4).

**Table 4:** Univariable and multivariable logistic regression analysis for significant liver fibrosis. OR: odds ratio; CI: confidence interval; BMI: body mass index; MASLD: metabolic dysfunction-associated steatotic liver disease. The adjusted model included sarcopenia as the primary exposure and age, sex, BMI, and diabetes mellitus as clinically relevant covariates.

Variable	Crude OR (95% CI)	p-value	Adjusted OR (95% CI)	p-value
Sarcopenia	2.97 (1.66-5.28)	<0.001	2.03 (1.10-3.77)	0.024
Age (per 10-year increment)	1.54 (1.21-1.95)	<0.001	1.37 (1.05-1.78)	0.020
Male sex (vs female)	0.73 (0.44-1.22)	0.233	0.76 (0.44-1.31)	0.321
BMI (per 5 kg/m² increment)	1.77 (1.22-2.58)	0.003	1.51 (1.00-2.29)	0.053
Diabetes mellitus	2.16 (1.29-3.63)	0.003	1.35 (0.75-2.43)	0.310
Hypertension	1.27 (0.77-2.12)	0.351	—	—
Dyslipidemia	1.55 (0.93-2.59)	0.091	—	—
Physical inactivity	1.72 (1.02-2.88)	0.041	—	—
MASLD duration (per year)	1.06 (0.98-1.15)	0.154	—	—

## Discussion

The present study found sarcopenia in 64 of 276 patients with MASLD, giving a prevalence of 23.2%. Among patients with sarcopenia, 33 (51.6%) were male, and 31 (48.4%) were female; sex did not differ significantly by sarcopenia status. Patients with sarcopenia were older, more often had diabetes and hypertension, and were less physically active. They also had higher HbA1c, fasting insulin, HOMA-IR, triglycerides, body fat, and visceral fat area. They also had higher CAP and liver stiffness, and LSM-defined significant fibrosis was more frequent (51.6% vs 26.4%). After adjustment for age, sex, BMI, and diabetes, sarcopenia remained statistically associated with LSM-defined significant fibrosis (adjusted OR 2.03, 95% CI 1.10-3.77).

The prevalence closely matches the pooled MASLD estimate of 23.5% from 29 studies involving 63,330 patients [5]. A nearly identical value was reported in 521 patients with biopsy-confirmed NAFLD, where low skeletal muscle mass was present in 23.4% [14]. This similarity may result from the recruitment of patients with a substantial metabolic burden and a history of fat-liver disease. In population-based metabolic dysfunction-associated fatty liver disease (MAFLD) samples from Korea and younger to middle-aged US people, lower prevalences of 9.9% and 12.75%, respectively, were found [14,15]. Those populations were younger or healthier and used relative appendicular muscle measures, whereas the present study incorporated muscle quantity, strength, and physical performance. Different associations have also been demonstrated when muscle mass was adjusted for body weight or BMI [16].

This similarity may result from the recruitment of patients with a substantial metabolic burden and a history of fat-liver disease. In population-based metabolic dysfunction-associated fatty liver disease (MAFLD) samples from Korea and younger to middle-aged US people, lower prevalences of 9.9% and 12.75%, respectively, were found [17,18]. Those populations were younger or healthier and used relative appendicular muscle measures, whereas the present study incorporated muscle quantity, strength, and physical performance. Different associations have also been demonstrated when muscle mass was adjusted for body weight or BMI [19].

Diabetes was more frequent, and HbA1c, fasting insulin, and HOMA-IR were higher among patients with sarcopenia. The strongest association with low muscle mass in one Korean study occurred in the diabetic MAFLD subgroup, supporting the importance of poor glucose control in this pattern [19]. Older age also increases susceptibility to muscle loss, while metabolic-risk-defined fatty liver has shown a higher sarcopenia burden than non-metabolic fatty liver [20]. Physical activity was markedly lower in the sarcopenia group. Among 11,690 patients with NAFLD, activity above 600 metabolic equivalent task (MET)-min/week was associated with lower odds of sarcopenia and fibrosis [21]. A similar inverse association was reported in U.S. data on NAFLD [22]. Higher hs-CRP may also be relevant, as inflammation partly mediated the MAFLD-sarcopenia association in a recent analysis [18].

LSM-defined significant fibrosis was more frequent in patients with sarcopenia than in those without sarcopenia (51.6% vs 26.4%). A biopsy-based study reported histologically significant fibrosis in 45.1% of patients with low skeletal muscle mass versus 30.8% without it, with an OR of 2.17 for sarcopenia [14]. These biopsy-based findings are not directly equivalent to the LSM-based fibrosis definition used in the present study. Transient elastography data also showed greater significant and advanced fibrosis when NAFLD and sarcopenia coexisted [6]. Long-term National Health and Nutrition Examination Survey (NHANES) data found more than twofold higher odds of significant fibrosis when MAFLD and sarcopenia occurred together [7], while recent Chinese and US MASLD populations showed an inverse relationship between ASMI and fibrosis severity after adjustment [9]. Earlier biopsy-based work also linked sarcopenia with nonalcoholic steatohepatitis (NASH) and fibrosis independent of obesity and insulin resistance [23]. In contrast, a study restricted to early NAFLD found muscle attenuation, rather than muscle mass, to be more closely related to steatohepatitis and fibrosis progression [8]. These findings should be interpreted with attention to differences in the methods used to assess both muscle status and liver fibrosis. Previous studies have reported associations of visceral adiposity and sarcopenic obesity with adverse liver-related findings [24,25], but the present study did not examine the independent associations of body fat percentage or visceral fat area with fibrosis.

Patients with sarcopenia had higher body fat percentage and visceral fat area despite the use of BMI in the adjusted model. Because BMI does not distinguish adiposity from muscle mass, some residual confounding is possible. Lower muscle mass and strength have also been reported in Asian MAFLD populations without low body weight [24]. The present findings show an association between sarcopenia and higher liver stiffness, but they do not establish that muscle assessment improves risk stratification or clinical outcomes. Further prospective studies are needed to determine whether muscle assessment adds clinical value in MASLD.

Strengths include the a priori sample-size calculation, consecutive recruitment, assessment of muscle quantity, strength and physical performance using AWGS 2019 criteria, detailed metabolic and body-composition measurements, objective CAP and liver stiffness measurements, and multivariable adjustment.

Limitations

The cross-sectional design cannot determine whether sarcopenia precedes fibrosis or develops with more advanced metabolic liver disease. Hospital-based consecutive sampling may limit generalizability, while bioelectrical impedance is less precise than DXA or CT and cannot directly assess myosteatosis. BMI does not distinguish muscle mass from adiposity, and differences in body fat percentage and visceral fat area may have resulted in residual confounding. Residual confounding from other unmeasured factors, single-time-point measurements, and lack of longitudinal outcomes also remain. Prospective multicenter studies that include a standardized muscle assessment protocol and repeated liver tests are required to establish the temporal relation between sarcopenia and MASLD progression.

## Conclusions

Almost one in every four individuals included in this study met the criteria for sarcopenia. This group tended to be older, was more commonly affected by diabetes and hypertension, and reported lower levels of physical activity than those without sarcopenia. Liver stiffness was also greater in these patients, and significant fibrosis was seen more often. When age, sex, body mass index, and diabetes were considered together in the adjusted analysis, sarcopenia remained statistically associated with LSM-defined significant fibrosis. This study did not evaluate whether adding muscle assessment improves diagnostic accuracy, prognostic prediction, or treatment decisions in MASLD.

## References

[1] Rinella ME, Lazarus JV, Ratziu V (2023). A multisociety Delphi consensus statement on new fatty liver disease nomenclature. J Hepatol.

[2] (2024). EASL-EASD-EASO clinical practice guidelines on the management of metabolic dysfunction-associated steatotic liver disease (MASLD). J Hepatol.

[3] Tilg H, Petta S, Stefan N, Targher G (2026). Metabolic dysfunction-associated steatotic liver disease in adults: a review. JAMA.

[4] Gupta V, Krishnamoorthy A (2025). Skeletal muscle alterations in metabolic dysfunction-associated steatotic liver disease: a critical review of diagnostic, mechanistic, and therapeutic intersections. World J Gastroenterol.

[5] Li X, He J, Sun Q (2024). The prevalence and effects of sarcopenia in patients with metabolic dysfunction-associated steatotic liver disease (MASLD): a systematic review and meta-analysis. Clin Nutr.

[6] Harring M, Golabi P, Paik JM (2023). Sarcopenia among patients with nonalcoholic fatty liver disease (NAFLD) is associated with advanced fibrosis. Clin Gastroenterol Hepatol.

[7] Zhao Q, Yin Y, Deng Y (2023). Metabolic associated fatty liver disease and sarcopenia additively increase mortality: a real-world study. Nutr Diabetes.

[8] Hsieh YC, Joo SK, Koo BK (2023). Myosteatosis, but not sarcopenia, predisposes NAFLD subjects to early steatohepatitis and fibrosis progression. Clin Gastroenterol Hepatol.

[9] Zhang F, Liu L, Li W (2025). Correlation of sarcopenia with progression of liver fibrosis in patients with metabolic dysfunction-associated steatotic liver disease: a study from two cohorts in China and the United States. Nutr J.

[10] Karlas T, Petroff D, Sasso M (2017). Individual patient data meta-analysis of controlled attenuation parameter (CAP) technology for assessing steatosis. J Hepatol.

[11] (2018). EASL clinical practice guidelines for the management of patients with decompensated cirrhosis. J Hepatol.

[12] (2024). KDIGO 2024 clinical practice guideline for the evaluation and management of chronic kidney disease. Kidney Int.

[13] Heidenreich PA, Bozkurt B, Aguilar D (2022). 2022 AHA/ACC/HFSA Guideline for the Management of Heart Failure: a report of the American College of Cardiology/American Heart Association joint committee on clinical practice guidelines. Circulation.

[14] Hsieh YC, Joo SK, Koo BK, Lin HC, Kim W (2021). Muscle alterations are independently associated with significant fibrosis in patients with nonalcoholic fatty liver disease. Liver Int.

[15] Chen LK, Woo J, Assantachai P (2020). Asian Working Group for Sarcopenia: 2019 consensus update on sarcopenia diagnosis and treatment. J Am Med Dir Assoc.

[16] Malmstrom TK, Morley JE (2013). SARC-F: a simple questionnaire to rapidly diagnose sarcopenia. J Am Med Dir Assoc.

[17] Chun HS, Kim MN, Lee JS (2021). Risk stratification using sarcopenia status among subjects with metabolic dysfunction-associated fatty liver disease. J Cachexia Sarcopenia Muscle.

[18] Feng Z, Zhao F, Wang Z, Tang X, Xie Y, Qiu L (2024). The relationship between sarcopenia and metabolic dysfunction-associated fatty liver disease among the young and middle-aged populations. BMC Gastroenterol.

[19] Seo JY, Cho EJ, Kim MJ (2022). The relationship between metabolic dysfunction-associated fatty liver disease and low muscle mass in an asymptomatic Korean population. J Cachexia Sarcopenia Muscle.

[20] Han E, Chun HS, Lee YH (2023). MAFLD might be better in identifying subjects with sarcopenia or cardiovascular risk than NAFLD: a nationwide study. J Gastroenterol Hepatol.

[21] Chun HS, Lee M, Lee HA (2023). Association of physical activity with risk of liver fibrosis, sarcopenia, and cardiovascular disease in nonalcoholic fatty liver disease. Clin Gastroenterol Hepatol.

[22] Golabi P, Gerber L, Paik JM, Deshpande R, de Avila L, Younossi ZM (2020). Contribution of sarcopenia and physical inactivity to mortality in people with non-alcoholic fatty liver disease. JHEP Rep.

[23] Koo BK, Kim D, Joo SK (2017). Sarcopenia is an independent risk factor for non-alcoholic steatohepatitis and significant fibrosis. J Hepatol.

[24] Choi KY, Kim TY, Chon YE (2023). Impact of anthropometric parameters on outcomes in Asians with metabolic dysfunction-associated fatty liver disease. J Cachexia Sarcopenia Muscle.

[25] Chun HS, Lee M, Lee HA (2023). Risk stratification for sarcopenic obesity in subjects with nonalcoholic fatty liver disease. Clin Gastroenterol Hepatol.

